# Identification of Overexpressed Genes in Malignant Pleural Mesothelioma

**DOI:** 10.3390/ijms22052738

**Published:** 2021-03-08

**Authors:** Federica Morani, Luisa Bisceglia, Giulia Rosini, Luciano Mutti, Ombretta Melaiu, Stefano Landi, Federica Gemignani

**Affiliations:** 1 Department of Biology, University of Pisa, 56126 Pisa, Italy; federica.morani@biologia.unipi.it (F.M.); luisa.bisceglia@student.unisi.it (L.B.); giulia.rosini@student.unisi.it (G.R.); ombretta.melaiu@unipi.it (O.M.); federica.gemignani@unipi.it (F.G.); 2 Center for Biotechnology, Sbarro Institute for Cancer Research and Molecular Medicine, College of Science and Technology, Temple University, Philadelphia, PA 19122, USA; chairman@gime.it; 3 Paediatric Haematology/Oncology Department, Ospedale Pediatrico Bambino Gesù, 00146 Rome, Italy

**Keywords:** malignant pleural mesothelioma, MPM, RNAseq, gene signature, overexpressed genes, therapeutic targets

## Abstract

Malignant pleural mesothelioma (MPM) is a fatal tumor lacking effective therapies. The characterization of overexpressed genes could constitute a strategy for identifying drivers of tumor progression as targets for novel therapies. Thus, we performed an integrated gene-expression analysis on RNAseq data of 85 MPM patients from TCGA dataset and reference samples from the GEO. The gene list was further refined by using published studies, a functional enrichment analysis, and the correlation between expression and patients’ overall survival. Three molecular signatures defined by 15 genes were detected. Seven genes were involved in cell adhesion and extracellular matrix organization, with the others in control of the mitotic cell division or apoptosis inhibition. Using Western blot analyses, we found that ADAMTS1, PODXL, CIT, KIF23, MAD2L1, TNNT1, and TRAF2 were overexpressed in a limited number of cell lines. On the other hand, interestingly, CTHRC1, E-selectin, SPARC, UHRF1, PRSS23, BAG2, and MDK were abundantly expressed in over 50% of the six MPM cell lines analyzed. Thus, these proteins are candidates as drivers for sustaining the tumorigenic process. More studies with small-molecule inhibitors or silencing RNAs are fully justified and need to be undertaken to better evaluate the cancer-driving role of the targets herewith identified.

## 1. Introduction

Malignant pleural mesothelioma (MPM) is a rare cancer of the pleura caused by a past exposure to asbestos. The patients’ median overall survival (OS) is <1 year with a 5-year survival rate <5% [1]. To date, beyond surgery, the combination of pemetrexed with cisplatin is the only clinically approved first-line chemotherapy, but it improves the OS by only 12.1 months [2,3]. Therefore, it is urgent to identify novel targets for future therapies, in the hope of improving patients’ survival and their quality of life. In the attempt to detect genes that play a role in determining the malignant phenotype and that could be exploited as possible therapeutic targets, many studies were carried out with the use of microarrays. These tools allow the parallel measurement of the transcriptome in a single experiment and, theoretically, they could allow the definition of a minimal set of deregulated genes relevant in the carcinogenesis process [4,5,6,7]. However, the practical experience showed a large interstudy variability with the definition of different deregulated genes according to different sample settings, methods of investigation, and analysis. Thus, there is a poor consistency among published studies and the obtained results have a limited robustness, creating a need for more research. Recently, in an effort to improve results from previous studies [6,8,9], Bai et al. applied computational analyses to gene-expression profiling data, validated with RNAseq, to identify MPM-specific, differentially expressed genes ending with a five-gene molecular signature that improved the risk stratification of MPM patients [10].

In order to detect novel targets for MPM, we carried out a computational analysis on RNAseq data from MPM tissues of 85 patients within The Cancer Genome Atlas (TCGA) database. The data were filtered and guided by patients’ prognostic information and by the results from published literature, allowing us to consolidate past results with novel investigations. Thus, a total of 15 overexpressed genes, the candidate drivers of MPM progression, have been detected. It is conceivable that if the overexpression of a given gene is relevant for driving and maintaining the malignant condition, this state also should be preserved when the primary tumor is cultivated in vitro. Moreover, once the tumor elicited permanent cell lines, the overexpressed state should be maintained indefinitely, at least for some of the genes. Then, in order to sort driver genes from the passenger ones, we analyzed in vitro the expression of the 15 proteins encoded by the detected genes in 6 MPM permanent cell lines (Mero-14, Mero-41, Mero-95, ZL-55, REN, and MSTO) and in 1 nonmalignant cell line (Met-5A). Our findings might provide specific biomarkers for prognosis and novel putative therapeutic targets for MPM.

## 2. Results

### 2.1. Identification of Differentially Expressed Genes (DEGs)

**Figure 1:**
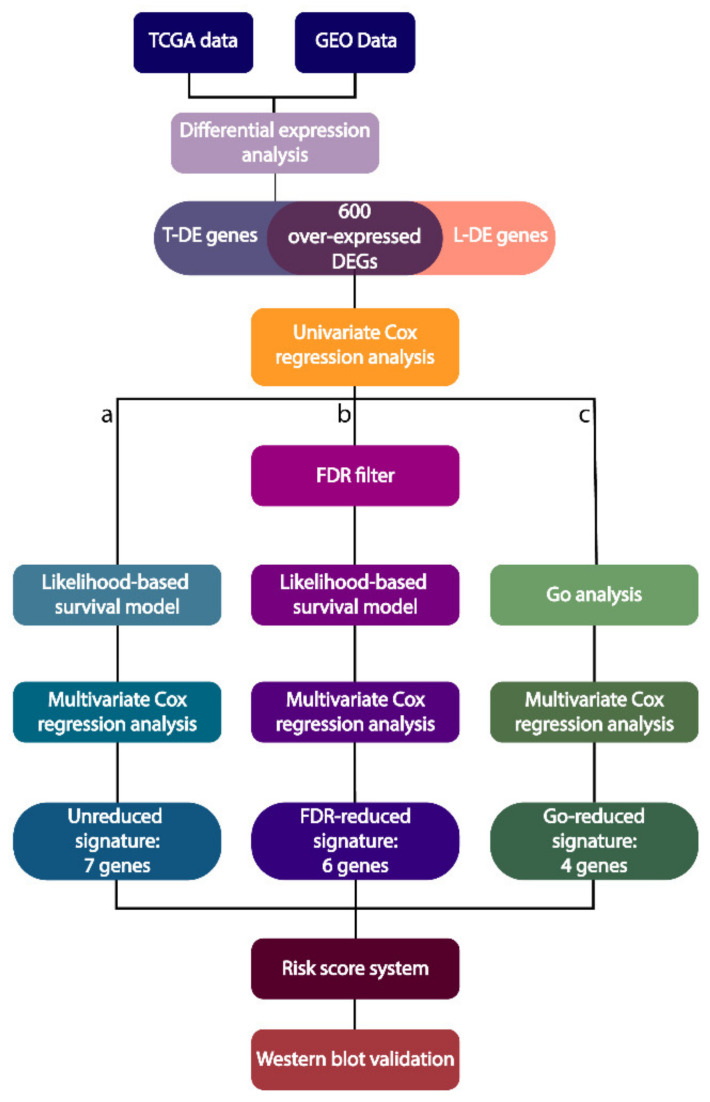
Flowchart of the DEG selection process.

The selection process for identifying the relevant DEGs is reported in detail in Figure 1.

**Figure 2:**
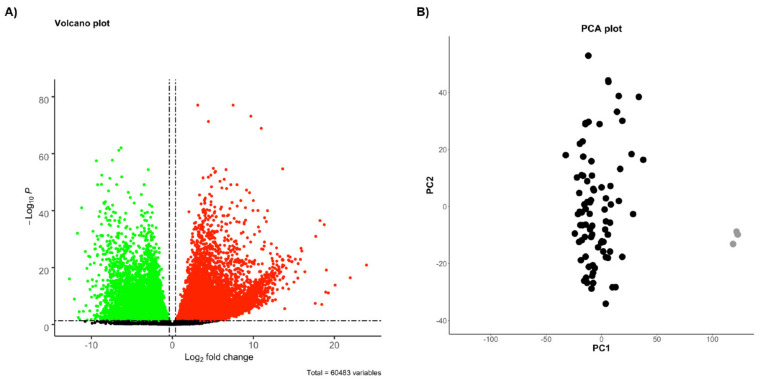
(**A**) Volcano plot. Cut-off criteria: *p*-value < 0.05 and |log2FC| > 0.38. The differentially expressed genes are in red (high-expressed) and in green (low-expressed), while the insignificantly changed genes are in black. (**B**) Principal component analysis (PCA) plot of 85 MPM patients (in black) and 3 normal lung samples (in grey). The PCA score plot showed that samples from MPM patients and controls were clustered separately.

Firstly, RNAseq data from healthy pleural tissues are not present in the available online resources. Thus, in order to obtain a list of statistically significant, differentially expressed (DE) genes of MPM, we intersected RNAseq-based transcriptomic data of MPM tissues obtained from TCGA dataset (*n* = 85) with the RNAseq data from three normal lung samples (*n* = 3) available in the Gene Expression Omnibus (GEO). We are aware that this type of reference sample was not optimal; however, this step was only used for a first analysis for a mild reduction of the number of genes. Thus, we identified 18,048 TCGA-derived DE genes (T-DE: 9536 high-expressed and 8512 low-expressed) in tumor samples (|FoldChange| > 1.3, and *p*-value < 0.05), as shown in the volcano plot in Figure 2.

**Figure 3:**
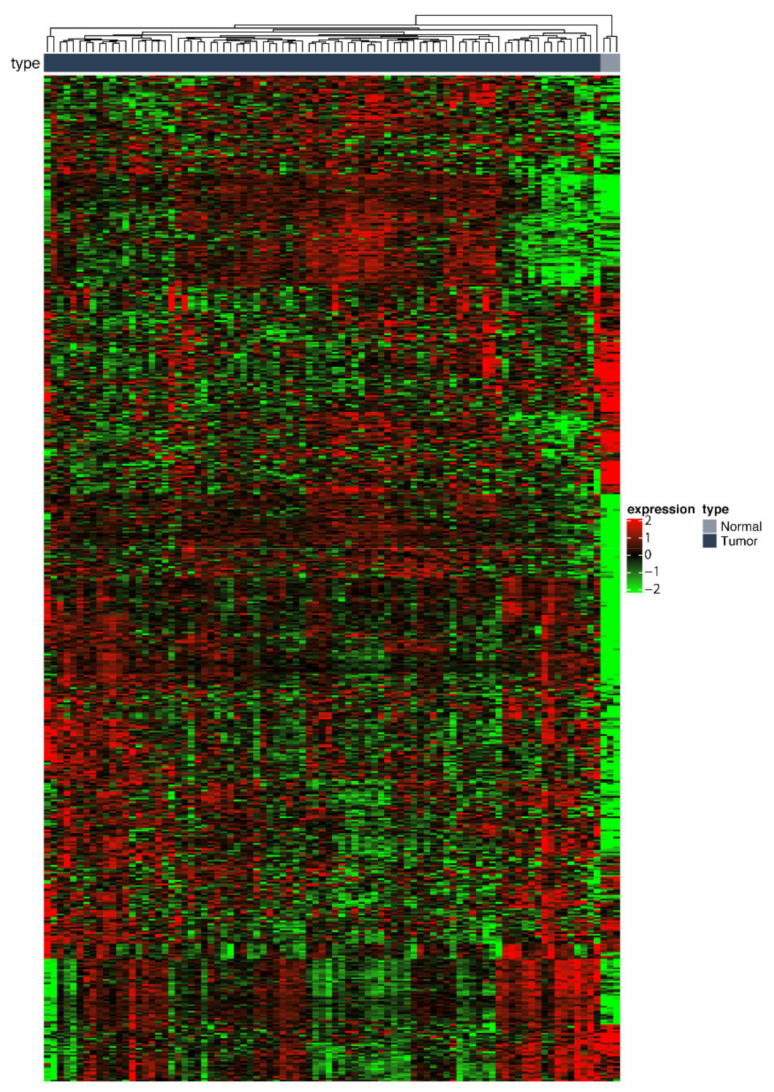
Heatmap and one-dimensional hierarchical clustering of the 839 filtered differentially expressed genes (DEGs) across MPM patients and a group of 3 nonmalignant lung samples. The genes are displayed in rows and samples are displayed in columns. High-expressed genes are in red; low-expressed genes are in green.

It should be considered that these genes do not necessarily describe the signature of a malignant state of the pleural tissue, but they could be normally overexpressed in healthy mesotheliocytes (as compared to the lung tissue) or in several types of cancers not limited to MPM. Thus, this broad list of T-DE genes was refined by the use of the manuscripts published from 2001 to date. By looking the literature, we selected genes showing at least one evidence of being differentially expressed in MPM, compared to nonmalignant pleura or nonmesothelioma cancer (the genes from the literature are defined as L-DE genes). In carrying out this step, we did not limit the positive selection to the main hits reported by the various authors. Rather, we went deep into the Supplementary Materials available for this study and selected genes showing any extent of differential expression, provided it was statistically significant. Thus, we intersected the T-DE list with the 1155 detected L-DE genes (T-DE ∩ L-DE), ending with 839 DE genes (600 high- and 239 low-expressed; Table S1), hereafter acronymized as DEGs. The 839 DEGs are reported in the heatmap in Figure 3.

### 2.2. Molecular Signatures Associated with the OS

In order to detect relevant targets for MPM, first we focused our attention on the genes whose extent of overexpression could correlate with a reduced patients’ OS. In fact, in view of identifying actionable targets for future therapies, inhibitors are easier to be designed as compared to gene activators. Thus, we carried out a survival analysis using a univariate Cox proportional hazard regression model on the 600 overexpressed genes ending, with 133 DEGs showing a nominal *p*-value < 0.05 and hazard ratio (HR) > 1 (Table S2). Then, the list of 133 DEGs was further reduced by using the unique criterion of correlating the extent of overexpression with the patients’ prognosis. In this case, two different approaches were employed: (a) the unreduced; or (b) the FDR-reduced.

For (a), the 133 DEGs were further refined by applying a robust likelihood-based survival modeling, and the Akaike information criterion (AIC) values showed 43 DEGs associated with OS. This list was further reduced by applying the multivariate Cox proportional regression analysis ending with seven DEGs positively associated with the OS: *CIT, KIF23, PODXL, PRSS23, SPARC, TRAF2,* and *UHRF1* (Table 1A).

For (b), the *p*-values of the univariate Cox proportional hazard regression model tests were corrected for multiple testing using Benjamini’s false-discovery rate (FDR) method. Thus, the 133 DEGs associated with the OS at the nominal value of *p* < 0.05 were reduced to 114. Next, we ran the robust likelihood-based survival modeling followed by the multivariate Cox proportional regression analysis, ending with six DEGs: *ADAMTS1, BAG2, KIF23, MAD2L1, TNNT1,* and *UHRF1* (Table 1B).

### 2.3. Molecular Signature Following a Functional Enrichment Analysis and Associated with the OS

**Figure 4:**
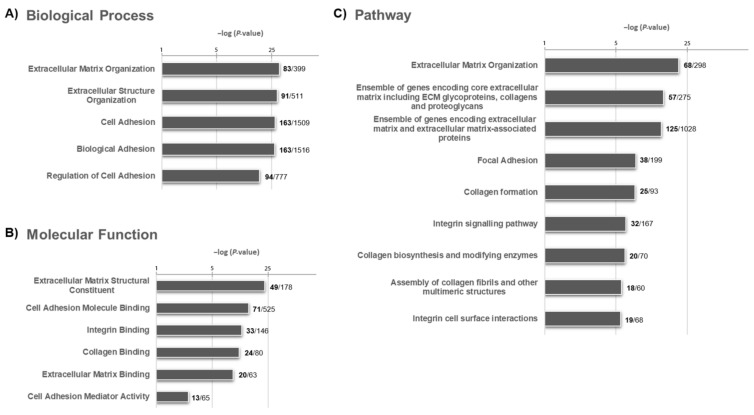
Bar plots showing significantly enriched GO terms associated with the (**A**) biological process, (**B**) molecular function, and (**C**) pathway categories in MPM. The number of DEGs for each term is indicated in bold alongside.

The list of 839 genes was also refined by applying a preliminary selection based on a functional enrichment assay with the software ToppFun, and a high share of DEGs (288) was shown to belong to the gene ontology (GO) biological process, molecular function, and pathway categories predominantly involved in cell adhesion and extracellular matrix (ECM) organization (*p*-value < 0.05). The detailed results of the enrichment analysis are reported in Figure 4.

Then, similarly to the analyses carried out before (a) and (b), we focused on the 600 overexpressed DEGs, and 225 were found to belong to these fields (Table S3). Thus, out of the 133 DEGs derived from the univariate COX regression analysis, 46 belonged to the above-mentioned functional pathways. These were directly used as input in the multivariate Cox proportional regression analysis, and 4 DEGs were found associated with the prognosis constituting a GO-reduced molecular signature (c): *CTHRC1, DSC3, MDK*, and *SELE* (Table 1C).

### 2.4. Construction of a Prognostic Risk Scoring System Using the Three Molecular Signatures

The molecular signatures extracted with the different approaches were evaluated for their strength of correlation with patients’ OS through the construction of a prognostic risk-scoring system. This was developed using the multivariate Cox proportional hazard regression coefficients and the degree of gene expression. Thus, the survival risk score (RS) for each patient was calculated as follows:

RS unreduced=2.033×CIT+ 3.943×KIF23+ 0.744×PODXL+1.198×PRSS23+ 1.399×SPARC+3.263×TRAF2+3.83×UHRF1 

RS FDR−reduced=0.681×ADAMTS1 + 1.390×BAG2+ 1.39×KIF23+ 1.847× MAD2L1+0.6271× TNNT1+1.353×UHRF1

RS GO−reduced=1.686×CTHRC1+0.863×DSC3+1.455×MDK+1.094×SELE

**Figure 5:**
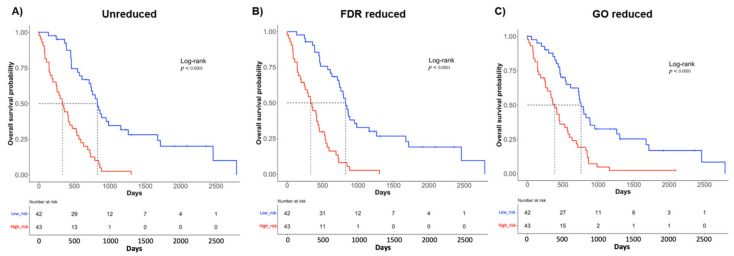
Kaplan-Meier (KM) survival curve of samples divided into high- (red) and low-risk (blue) groups according to the median (log-rank test *p*-value < 0.0001). (**A**) KM of unreduced gene signature; (**B**) KM of FDR-reduced gene signature; (**C**) KM of GO-reduced gene signature.

Then, we stratified the samples into a high-risk group (43 MPM samples) and a low-risk group (42 MPM samples) according to the median of the 3 risk-scores, and we carried out a survival analysis by plotting the Kaplan-Meier curves. As expected (Figure 5), there was a statistically significant shorter OS in high-risk patients than in low-risk patients (log-rank test *p*-value < 0.0001) for all the signatures.

**Figure 6:**
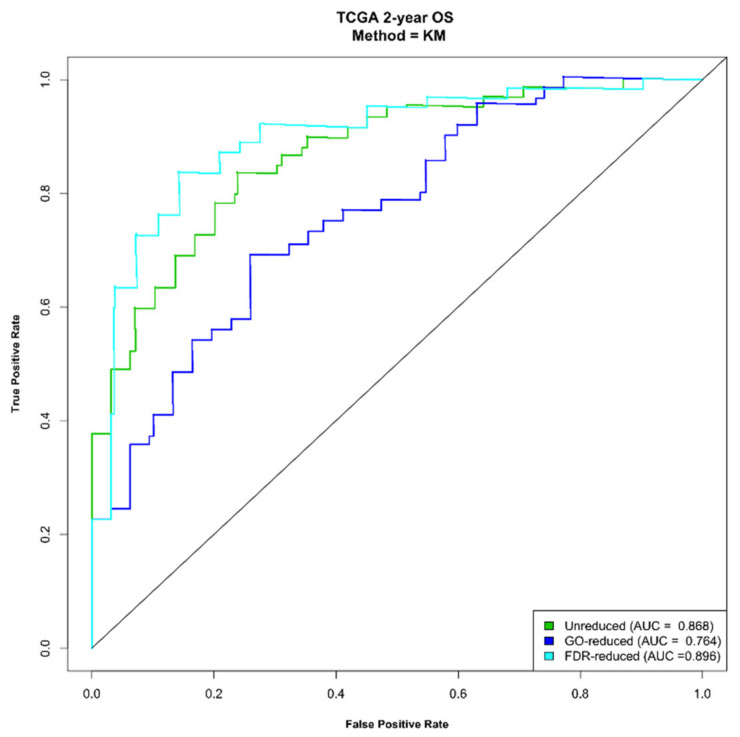
Time-dependent receiver operating characteristic (ROC) curve for predicting 2-year survival.

We used time-dependent receiver operating characteristic (ROC) curves and the area under the ROC curve (AUC) to evaluate the sensitivity and specificity of each signature. We found that the a and b signatures showed similar performances with AUC, ranging from 0.816 (for the 1-year OS, signature a) to 0.896 (for the 2-year OS, signature b). Signature c showed a slightly lower performance (AUC = 0.729 and 0.764 for 1- and 2-year OS, respectively). In Figure 6, the plots are reported for the OS at 2 years.

### 2.5. The Signatures Are Independent Prognostic Factors

The gene-signature risk scores were evaluated as covariates, together with other patient parameters, including age, gender, stage, and histological type. The univariate Cox regression analysis showed that the histological type of the tumor and the risk scores of the three signatures were the only covariates associated with OS in a statistically significant way (*p* < 0.05 and HR >1) (Table 2).

When evaluated in a multivariate model, RS for FDR-reduced and GO-reduced signatures remained the only statistically significant covariate, suggesting an independent prognostic factor for MPM (Table 3).

### 2.6. In Vitro Validation of the Prognostic Signatures by Protein Analysis of MPM Cell Lines

**Figure 7:**
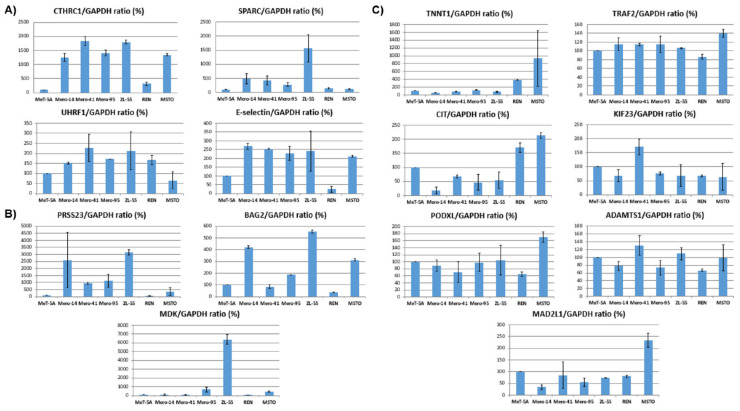
The densitometry ratios of proteins encoded by genes obtained from in silico analyses normalized versus GAPDH. (**A**) Proteins overexpressed in at least 5 MPM cell lines; (**B**) proteins overexpressed in 4 or 3 MPM cell lines; (**C**) proteins overexpressed in only 2 or 1 MPM cell lines. Data shown in this figure were reproduced independently 3 times. Corresponding blots are reported in Figure S1 and Supplementary File S1.

The three signatures defined a pool of 15 overexpressed DEGs likely relevant for driving and maintaining the malignant state of MPM cells. Thus, in order to validate these findings, the expression levels of the encoded proteins were evaluated in vitro by the use of Western blotting. We analyzed the nonmalignant cell line Met-5A and the MPM cell lines ZL-55, REN, MSTO, Mero-14, Mero-41, and Mero-95 for the expression of SPARC, CIT, TRAF2, PODXL, KIF23, PRSS23, UHRF1, E-SELECTIN, CTHRC1, MDK, ADAMTS1, DSC3, TNNT1, BAG2, and MAD2L1. Relative to Met-5A, CTHRC1 was overexpressed in all MPM cell lines, while E-selectin, SPARC, and UHRF1 were overexpressed in five out of six MPM cell lines (Figure 7A). Compared to Met-5A, PRSS23 and BAG2 were abundantly expressed in 4 MPM cell lines, whereas MDK was in 3 MPM cell lines (Figure 7B). The remaining seven proteins (ADAMTS1, PODXL, CIT, KIF23, MAD2L1, TNNT1, and TRAF2) were found at high levels in only two or one MPM cell lines (Figure 7C). Finally, DSC3 was not detectable in our cellular models (data not shown). Representative blots are reported in Figure S1.

## 3. Discussion

In an effort to identify actionable targets for MPM, the detection of overexpressed DEGs is of pivotal importance. In the present work, we began with the RNAseq data of 85 MPM patients available in TCGA. Then, we carried out a differential expression analysis followed by a comparison with the published literature, yielding a gene list of 839 DEGs enriched with MPM-specific genes. The application of statistical models based on the patients’ OS and a GO-enrichment ended with the computation of 3 molecular signatures associated with the OS and identification of 15 genes. The computational methods allowed cross-validation, which is essential in predictive modeling for data with large variability. These classifiers could successfully identify two groups of MPM patients (high-/low-risk) associated with significant differences in OS. Furthermore, a multivariate Cox regression analysis suggested that the molecular signatures were also independent prognostic factors from other clinical parameters such as age at diagnosis, stage, and histology. In this study, the epithelioid was confirmed to be the histotype with longer OS compared to the other histotypes. Nevertheless, the signatures could discriminate patients on the basis of their OS at a molecular level and showed to be a more robust marker than age or stage. Present findings may provide novel specific biomarkers for prognosis and could have significant implications in the understanding of therapeutic targets for MPM.

Despite showing that the increased expression of the 15 genes was associated with the OS, it is still unknown whether the overexpression of these DEGs is a driver or a passenger of the malignant state. In the attempt to shed some light, we measured the expression of the encoded proteins by Western blot in a series of MPM cell lines. We are aware that cell lines are not fully representative of their tumor of origin. However, the rationale is that an overexpression of oncoproteins responsible for sustaining the malignant phenotype should be maintained, at least for some of them, in the derivative cell lines also after many passages of in vitro growth conditions. Of the 15 proteins, CTHRC1 was overexpressed in all MPM cell lines (relative to MeT-5A), while E-selectin, SPARC, and UHRF1 were in 5 out of 6. PRSS23 and BAG2 were abundantly expressed in four MPM cell lines; MDK in three MPM cell lines. According to our departing hypothesis, these proteins are likely important drivers for sustaining MPM tumorigenesis. On the other hand, the remaining seven proteins (ADAMTS1, PODXL, CIT, KIF23, MAD2L1, TNNT1, and TRAF2) were overexpressed in only two or one MPM cell lines, and this could be interpreted as a sign of a limited role in driving the malignancy. However, a high level of KIF23 and MAD2L1 has been found in the majority of MPM clinical cases, and it correlated with a poor OS. In addition, their increased expression also was found in several human MPM cell lines [11,12]. Interestingly, an increased expression of MDK, UHRF1, and SPARC was observed in MPM tissues as well, and it correlated with poor patients’ OS together with elevated expression in MPM cell lines [13,14,15]. Thus, all these observations are in agreement with our results and strongly suggest that at least CTHRC1, E-selectin, SPARC, UHRF1, PRSS23, BAG2, MDK, KIF23, and MAD2L1 could play an important role in MPM carcinogenesis as biomarkers of prognosis, and constitute novel therapeutic targets for MPMs.

With intent to understand the relevance of these proteins for MPM, we attempted to group them based on their molecular function. CTHRC1, E-selectin (encoded by *SELE*), MDK, and SPARC are known to be involved in cell adhesion and ECM. In detail, CTHRC1 (collagen triple helix repeat containing-1) is a cancer-related extracellular protein. It regulates multiple signaling pathways, promoting tumor development and metastasis [16]. Furthermore, in different microenvironments, CTHRC1 shows specific cellular localization and activity. A future detailed investigation on its role in vitro in our cellular models will be required to elucidate its putative role as biomarker for predicting tumor recurrence or metastasis in MPM. E-selectin, also called CD62E, is a cell adhesion protein located on endothelial cells activated by cytokines and involved in inflammation and in tumor cells adhesion to the endothelium. Since most of solid tumors over-express E-selectin, a large body of literature describes its potential use as therapeutic target. In particular, new immunoliposomes and nanocarrier systems for targeted delivery of rapamycin to TNF-α activated endothelial cells have been developed [17,18,19]. Despite none is known on whether E-selectin could be exploited for drug deliveries in MPM patients, present data are encouraging to undertake this direction. MDK (Midkine), a heparin-binding growth factor, is abnormally overexpressed in several human malignancies playing a key role during tumor development [20]. SPARC (secreted protein acidic and rich in cysteine, also known as osteonectin or BM-40) is a crucial protein for cell-cell interactions, ECM remodeling, and bone mineralization [21]. In the tumor microenvironment, it plays a role in tumor growth, metastasis formation, invasion, and EMT. SPARC is normally expressed by stromal cells, showing either tumor suppressor or pro-oncogenic functions according to different types of cancer [21]. In a recent study on MPM, Kao et al. used a proteomic-based approach to explore potential biomarkers in the plasma of MPM patients, and they found SPARC to be a circulating prognostic biomarker [15]. This is in agreement with our data obtained from the transcriptome analysis that reported an increased expression of the encoded protein correlating with shorter OS. However, the specific function of SPARC in MPM has not been clarified yet, and further studies are needed to understand the actual role of this protein in MPM tumorigenesis. The fact that SPARC protein was found to be upregulated in almost all the MPM cell lines analyzed in the present work strongly suggests that it is an important molecule for MPM progression, and more studies aimed to exploit it as therapeutic target should be undertaken.

Conversely, KIF23, MAD2L1, and UHRF1 were involved in mechanisms related to the control of mitotic cell division. In particular, KIF23 is a member of the kinesin motor protein involved in the regulation of cytokinesis [22,23]. KIF23 overexpression is a common event seen in various tumors, such as glioma [24], breast [25], and paclitaxel-resistant gastric cancer [26]. MAD2L1 (MAD2 mitotic arrest deficient-like 1) belongs to the mitotic spindle assembly checkpoint (MSAC) pathway. It is required during mitosis for preventing the start of anaphase when chromosomes are not properly aligned in the equatorial plate [27,28,29]. Dysregulation of MAD2L1 is associated with chromosomal instability and substantial aneuploidy, which frequently occurs in cancer cells [30]. UHRF1 (ubiquitin-like with plant homeodomain and ring finger domains 1) plays a major role in the G1/S transition, and its expression is high throughout the cell cycle, until the late M phase. Its role during cell division is not well characterized. However, it has been acknowledged as an important master epigenetic regulator of gene expression, in particular during mitosis and DNA repair, acting through DNA methylation and chromatin remodeling [31,32,33,34,35].

On a final note, BAG2 and PRSS23 showed an independent function that could not be linked to the others. In depth, BAG2 (Bcl2-associated athanogene 2) displayed an antiapoptotic role. It is a cochaperone with broad activities devoted to negatively regulate various cellular functions involved in the pathogenesis of several disorders ranging from cancers to neurodegenerative diseases. Among its targets, one of the most important is the heat shock protein 70 (Hsp70) [36]. In agreement with our study, BAG2 is frequently found to be upregulated in tumors, pointing out its ability to also promote proliferation and metastasis by promoting the accumulation of mutant p53 [37,38,39,40]. According to the results of our work and the knowledge on BAG2, studies evaluating strategies targeting BAG2 in the fight against MPM should be undertaken. PRSS23 instead belongs to the trypsin family of serine proteases. PRSS23 is reported to be a positive regulator of EMT [41,42]. Upregulated PRSS23 was associated with breast cancer cell proliferation [43], and PRSS23 knockdown could inhibit gastric cancer [44]. However, studies on these molecules are very limited in the context of cancer. Interestingly, in a recent study sharing analogies with our work, PRSS23 was found to be a component of an 18-gene molecular signature associated with the OS of patients affected by pancreatic ductal adenocarcinoma [45].

In the future, further analysis exploring the possibility of targeting these genes with novel therapeutic agents is fully justified. In fact, our findings suggest that the studied 15 proteins could constitute effective druggable targets for patients with MPM. Certainly, preliminary studies in vitro to investigate the specific gene function in MPM (e.g., gene silencing and functional tests) will be required before testing candidate drugs.

## 4. Materials and Methods

### 4.1. Public Databases

Transcriptomic data of MPM patients (*n* = 85) were obtained from TCGA, available online at: https://portal.gdc.cancer.gov/projects/TCGA (accessed on 25 March 2020). We integrated TCGA data with the RNAseq data from a GEO cohort of normal lung samples (*n* = 3), downloaded at the link: https://www.ncbi.nlm.nih.gov/geo/query/acc.cgi (accessed on 26 March 2020), as a control group (GSE94555).

### 4.2. Data Processing and Computational Analysis

RNA sequencing data for 85 patients with MPM and 3 samples from normal lung tissues were processed/reprocessed using the same pipeline described in the GDC (Genomic Data Commons Data Portal, https://portal.gdc.cancer.gov/, accessed on 25 March 2020), the data portal of TCGA. In brief, the downloaded data were analyzed with FASTQC [46], and then the processed reads were mapped to the human genome (GRCh38.d1.vd1) using STAR [47]. To obtain quantification scores for all human genes and transcripts across all samples, raw counts were calculated using HTSeq [48]. The genes ID was annotated to obtain the gene names, the biotype, and general information using the biomaRt package [49].

### 4.3. Differential Expression Analysis

The raw counts for the 85 MPM and 3 normal lung specimens were used as input for DESeq2 [50], allowing us to identify a set of genes differentially expressed (DE) in a statistically significant way, referred to here as T-DE genes. All data were processed and analyzed using R language available at: https://www.R-project.org (accessed on 15 March 2020).

### 4.4. Literature Search Strategy

All papers inclusive of transcriptomics data on MPM and reporting a complete list of DEGs were selected from literature. The search terms for studies selection on PubMed were the following: “transcriptome” AND “analysis” AND “mesothelioma”. A final list of genes was obtained from 13 eligible studies, published from 2001 to date [9,51,52,53,54,55,56,57,58,59,60,61,62], and referred to here as L-DE genes. In detail, the comparisons carried out in the eligible studies were: (a) Met-5A (an SV40 immortalized nonmalignant human mesothelial cell line) vs. MSTO-211H (one MPM cell line) [51]; (b) cell lines derived from 4 patients diagnosed with primary malignant mesothelioma vs. 2 primary mesothelial cell cultures from pleural fluid of 2 noncancer patients [55]; (c) mesothelioma tissue specimens from 16 patients vs. 4 control pleural tissue samples [56]; (d) 2 MPM primary tumors and the MSTO-211H cell line vs. MeT-5A [58]; (e) 11 mesothelioma cell lines and 4 primary tumor specimens vs. Met5A [57]; (f) 40 human MPM tumor specimens and 4 MPM cell lines vs. 4 normal lung sample, 5 normal pleura specimens, and Met-5A [9]; (g) 4 MPM samples vs. 1 normal lung sample [60]; (h) 5 tissues from mesothelioma patients vs. normal and visceral pleural samples from 6 noncancer patients [61,62]; (i) 61 MPM cell lines vs. 25 lung adenocarcinoma or 15 benign tumors from pleural effusions [54]; (l) 100 MPM specimens vs. 12 nonmalignant pleural samples [52]; (m) 6 epithelioid mesothelioma vs. 6 pulmonary adenocarcinoma [53]; (n) 15 effusions of diffuse peritoneal MM (*n* = 6) vs. ovarian carcinoma (*n* = 4)/primary peritoneal carcinoma (*n* = 5) [59]. In selecting L-DE genes, we did not limit the positive selection to the main hits reported by the various authors. Rather, we went deep into the Supplementary Materials available for this study and selected genes showing any extent of differential expression, provided it was statistically significant, and we considered the genes showing evidence of being differentially expressed in MPM in at least one study.

### 4.5. Functional Enrichment Analysis

Functional annotation enrichment analysis of GO relative to biological functions, biological processes, and pathways was performed assuming the statistical background of the whole genome. The input list of T-DE ∩ L-DE genes was introduced to the portal ToppFun, an application of the ToppGene Suite, available at: https://toppgene.cchmc.org/ (accessed on 30 April 2020). ToppFun reported functional enrichment analysis of an input gene list based on ontologies (GO, pathway). Functional enrichments were provided by the ToppFun algorithm, which employs hypergeometric distribution with multiple correction testing according to Benjamini’s FDR method [63] to determine statistical significance.

### 4.6. Gene Signature Identification

The identification of OS-related RNAs among the differentially expressed RNAs was carried out by employing the univariate Cox proportional hazards regression analysis (two-sided) and using the FDR for the calculation of the statistical significance. Hazard ratios (HRs) and 95% confident intervals (95% CIs) were calculated for each gene. Then, to obtain robust and survival-associated genes, we constructed a robust likelihood-based survival model using the rbsurv package [64]. All patients were randomly assigned to a training set with Nx(1-p) samples and a testing set with Nxp samples (p = 1/3). This package uses a forward gene selection procedure to assign a parameter for each gene and evaluated the log-likelihood with the parameter estimate and validation dataset. The above procedure was repeated 10 times, resulting in 10 log-likelihoods for each gene. The best gene with the largest mean log-likelihood was selected. The procedure was iterated 10 times. The output was further refined by applying a multivariate model of the Cox analysis (two-sided). The Cox proportional analysis was carried out by using survival R package [65]. Then, an RS was established for each patient by calculating the DESeq2-normalized expression values of the selected genes weighted by regression coefficients in the multivariate Cox regression analysis. The formula used was as follows:

$$\mathrm{Risk}\;\mathrm{score}=\;\overset{n}{\underset{i=1}{\sum }}\exp_{i}\times {\;\beta }_{i}$$

where *n* is the number of selected genes, expi is the expression level of gene *i* normalized by DESeq2, and β*_i_* represents the regression coefficient of gene *i*. Subsequently, the patients were divided into two groups: high-risk score and low-risk score, based on the risk score’s median. A ROC curve analysis was carried out to compare the predictive accuracy of the gene signature. A *p*-value < 0.05 was set as the statistically significant difference. Heatmaps were generated in R with the ComplexHeatmap package [66] with z-score normalization within each row.

### 4.7. Survival Analysis

Based on the median of each risk score, we classified the patients into two groups (high-risk and low-risk). The prognosis of each group of patients was examined by Kaplan-Meier survival estimators, and the survival outcomes of the two groups were compared by log-rank tests. The Kaplan-Meier analysis with the log-rank test for difference was performed with the R package survival.

### 4.8. Cell Culture

Normal human mesothelial cells Met-5A and MPM cell lines ZL-55, REN, and MSTO were obtained from American Type Culture Collection (ATCC, Manassas, VA, USA). MPM cell lines Mero-14, Mero-41, and Mero-95 were obtained from European Collection of Authenticated Cell Culture (ECACC, Porton Down, UK). Met-5A cells were grown in Medium 199 supplemented with 10% FBS, 3.3 nM EGF, 400 nM hydrocortisone, and 870 nM zinc-free bovine insulin (all from Gibco, Carlsbad, CA, USA). Mero-14, Mero-41, and Mero-95 cells were grown in HAMS F10; ZL-55 and MSTO cells were grown in a 1:1 mixture of DMEM and Ham’s F-12; REN cells were grown in DMEM (all from Euroclone S.p.A., Milan, Italy). All MPM cells were maintained in medium supplemented with 10–15% heat-inactivated FBS, 2mM L-glutamine, 100 U/mL penicillin, and 100 U/mL streptomycin (all from Euroclone S.p.A., Milan, Italy). Cells were kept at 37 °C in a constant humidified 5% CO_2_ atmosphere.

### 4.9. Western Blot Analysis

Cells were collected at confluence, washed twice with PBS, and then homogenized in Mammalian Protein Extraction Reagent (M-PER, ThermoFisher Scientific, Waltham, MA, USA) containing inhibitors of proteases and phosphatase (Roche Diagnostics GmbH, Rotkreuz, Switzerland), following standard protocols. Then, 7 μg of proteins, determined by BCA assay (Invitrogen-Life Technologies, Carlsbad, CA, USA), were denatured, separated by electrophoresis using precast Novex 8–16% or 4–12% Wedge Wells Tris-Glycine Gels (Invitrogen-Life Technologies, Carlsbad, CA, USA), and electroblotted onto PVDF membranes (Bio-Rad Laboratories Inc., Hercules, CA, USA). The membranes were blocked with 5% milk TBST and probed overnight at 4 °C with the specific primary antibody: anti-CIT rabbit polyclonal antibody (1:500; Proteintech, Rosemont, IL, USA); anti-CTHRC1 rabbit polyclonal antibody (1:1000; Proteintech, Rosemont, IL, USA); anti-E selectin rabbit polyclonal antibody (1:750; Proteintech, Rosemont, IL, USA); anti-Midkine (MDK) rabbit polyclonal antibody (1:500; Proteintech, Rosemont, IL, USA); anti-SPARC rabbit polyclonal antibody (1:750; Proteintech, Rosemont, IL, USA); anti-TRAF2 rabbit polyclonal antibody (1:1000; Proteintech, Rosemont, IL, USA); anti-UHRF1 rabbit polyclonal antibody (1:1000; Proteintech, Rosemont, IL, USA); anti-DSC3 mouse polyclonal antibody (1:500; Genetex, Irvine, CA, USA); anti-KIF23 rabbit polyclonal antibody (1:500; OriGene, Rockville, MD, USA); anti-PRSS23 rabbit polyclonal antibody (1:500; Abcam, Cambridge, MA, USA); anti-ADAMTS1 rabbit polyclonal antibody (1:500; GeneTex, Irvine, CA, USA); anti-PODXL rabbit polyclonal antibody (1:500; Proteintech, Rosemont, IL, USA); anti-BAG2 rabbit polyclonal antibody (1:1000; Proteintech, Rosemont, IL, USA); anti-TNNT1 rabbit polyclonal antibody (1:1000; Proteintech, Rosemont, IL, USA); anti-MAD2L1 rabbit polyclonal antibody (1:800; Proteintech, Rosemont, IL, USA). An antibody specific for GAPDH (1:10,000; Proteintech, Rosemont, IL, USA) was used as an index of homogenate protein loading in the lanes. Secondary antibodies anti-rabbit IgG-HRP (1:10,000; Jackson ImmunoResearch laboratories, West Grove, PA, USA) and anti-mouse IgG-HRP (1:20,000; Proteintech, Rosemont, IL, USA) were added for 1 h at room temperature and used for signal detection. Reactive bands were detected using Clarity MaxTM Western ECL Substrate (Bio-Rad Laboratories Inc., Hercules, CA, USA), according to the manufacturer’s instructions. Visualization was performed using a ChemiDoc Imaging System (Bio-Rad Laboratories Inc., Hercules, CA, USA). Densitometry of Western blot bands was carried out with the ImageLab 6.0 software (Bio-Rad Laboratories Inc., Hercules, CA, USA).

## 5. Conclusions

In summary, we identified an elevated expression of 15 genes in MPM tissues associated with a worse patient OS. Among them, seven also showed a high protein expression in the panel of MPM cell lines herewith analyzed, and two more were reported as overexpressed in other published studies on MPM cells. All these findings suggest that the identified molecules could be exploited as prognostic biomarkers and new therapeutic targets for MPM. A better understanding of the role of these putative biomarkers remains to be elucidated. To this end, further functional analyses in vitro on MPM cellular lines will be needed.

## Figures and Tables

**Table 1:** Multivariate Cox analysis of the three gene signatures: (**A**) unreduced; (**B**) FDR-reduced; (**C**) GO-reduced.

**(A)**											
				**Multivariate Cox Results**							
**Symbol**	**Ensembl ID**			**Coef**	**HR**	**Se(Coef)**			**Z**		***p*** **-Value**
*SPARC*	ENSG00000113140.9			1.40	4.05	0.53			2.63		8.44 × 10^−3^
*CIT*	ENSG00000122966.12			2.03	7.64	0.65			3.11		1.9 × 10^−3^
*TRAF2*	ENSG00000127191.16			3.26	26.13	0.97			3.36		7.86 × 10^−4^
*PODXL*	ENSG00000128567.15			0.74	2.10	0.32			2.29		2.2 × 10^−2^
*KIF23*	ENSG00000137807.12			3.94	51.57	1.19			3.31		9.21 × 10^−4^
*PRSS23*	ENSG00000150687.10			1.20	3.31	0.35			3.44		5.86 × 10^−4^
*UHRF1*	ENSG00000276043.3			3.83	46.05	0.99			3.88		1.04 × 10^−4^
**(B)**											
			**Multivariate Cox Results**								
**Symbol**		**Ensembl ID**	**Coef**		**HR**		**Se(Coef)**	**Z**		***p*** **-Value**	
*MAD2L1*		ENSG00000164109.12	1.85		6.34		0.40	4.68		2.93 × 10^−6^	
*KIF23*		ENSG00000137807.12	1.39		4.02		0.67	2.08		3.75 × 10^−2^	
*UHRF1*		ENSG00000276043.3	1.35		3.87		0.52	2.62		8.86 × 10^−3^	
*ADAMTS1*		ENSG00000154734.13	0.68		1.98		0.24	2.89		3.86 × 10^−3^	
*TNNT1*		ENSG00000105048.15	0.63		1.87		0.23	2.78		5.43 × 10^−3^	
*BAG2*		ENSG00000112208.11	0.40		1.49		0.17	2.39		1.71 × 10^−2^	
**(C)**											
			**Multivariate Cox Results**								
**Symbol**		**Ensembl ID**	**Coef**		**HR**		**Se(Coef)**		**Z**		***p*** **-Value**
*MDK*		ENSG00000110492.14	1.46		4.29		0.40		3.60		3.17 × 10^−4^
*SELE*		ENSG00000007908.14	1.09		2.99		0.33		3.29		9.88 × 10^−4^
*CTHRC1*		ENSG00000164932.11	1.69		5.40		0.70		2.40		1.66 × 10^−2^
*DSC3*		ENSG00000134762.15	0.86		2.37		0.39		2.23		2.57 × 10^−2^

**Table 2:** Univariate survival analysis concerning clinical parameters. HR, hazard ratio; RS, risk score.

Variable	Beta	HR (95% CI for HR)	Wald Test	*p*-Value
Age at index median	0.29	1.3 (0.83–2.1)	1.5	0.23
Primary diagnosis: epithelioid vs. other	−0.64	0.53 (0.32–0.86)	6.4	0.01
Primary diagnosis: biphasic vs. other	0.63	1.9 (1.1–3.2)	5.3	0.02
Gender: male vs. female	−0.008	0.99 (0.54–1.8)	0	0.98
Tumor stage	−0.036	0.96 (0.58–1.6)	0.02	0.89
RS: unreduced median	1.4	4 (2.4–6.7)	28	1.2 × 10^−7^
RS: FDR-reduced median	1.5	4.3 (2.6–7.2)	31	2.6 × 10^−8^
RS: GO-reduced median	0.98	2.7 (1.6–4.3)	16	7.6 × 10^−5^

**Table 3:** Multivariate survival analysis concerning clinical parameters. HR, hazard ratio; RS, risk score; se, standard error.

Variable	Beta	HR	Se(Coef)	Z	*p*-Value
Age at index median	0.192	12.122	0.268	0.718	0.472
Primary diagnosis: epithelioid vs. other	−0.625	0.535	0.463	−1.352	0.176
Primary diagnosis: biphasic vs. other	−0.280	0.756	0.512	−0.547	0.584
Gender: male vs. female	−0.480	0.619	0.342	−1.405	0.160
Tumor stage	−0.317	0.728	0.282	−1.124	0.261
RS: unreduced median	0.683	19.800	0.591	1.156	0.248
RS: FDR-reduced median	12.228	33.967	0.469	2.607	0.009
RS: GO-reduced median	0.980	26.655	0.279	3.520	4 × 10^−4^

## Data Availability

Publicly available datasets were analyzed in this study. This data can be found here: TCGA, project: TCGA-MESO, https://portal.gdc.cancer.gov/projects/TCGA (accessed on 25 March 2020); GEO, accession number: GSE94555, https://www.ncbi.nlm.nih.gov/geo/query/acc.cgi (accessed on 26 March 2020). References are given in the text for the datasets taken from the literature on MPM. The new data presented in this study are available in Results section and the Supplementary Materials.

## Supplementary Materials

The following are available online at https://www.mdpi.com/1422-0067/22/5/2738/s1.
