# Supplementary figures and images for: Identification of Overexpressed Genes in Malignant Pleural Mesothelioma
Source: Int J Mol Sci. 2021 Mar 8;22(5):2738. doi: 10.3390/ijms22052738 (PMC7962966; doi:10.3390/ijms22052738)

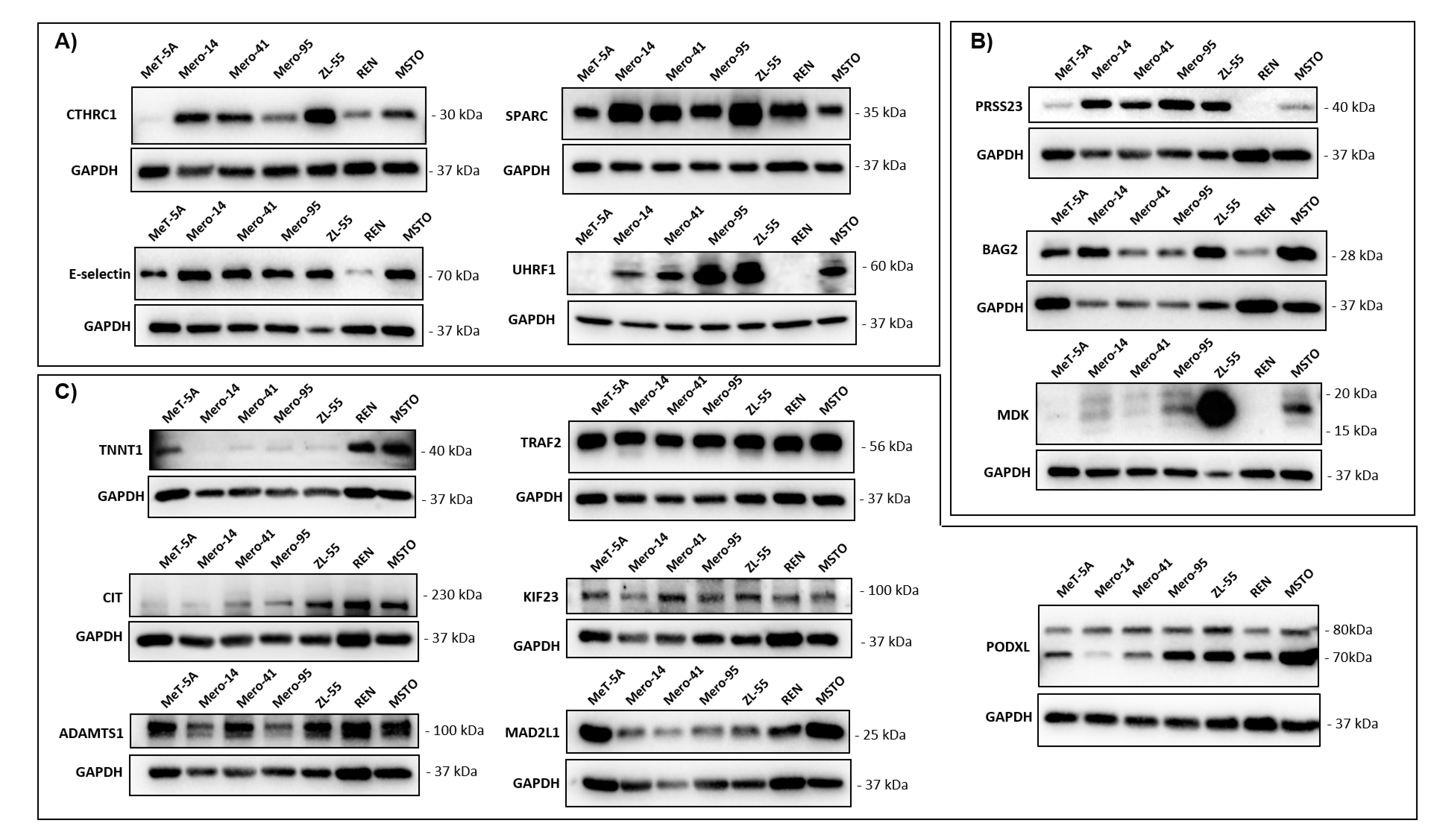

Supplement: Supplementary file 1 [file ijms-22-02738-s001.zip › Supplementary_final/Supplementary figure S1.tif]

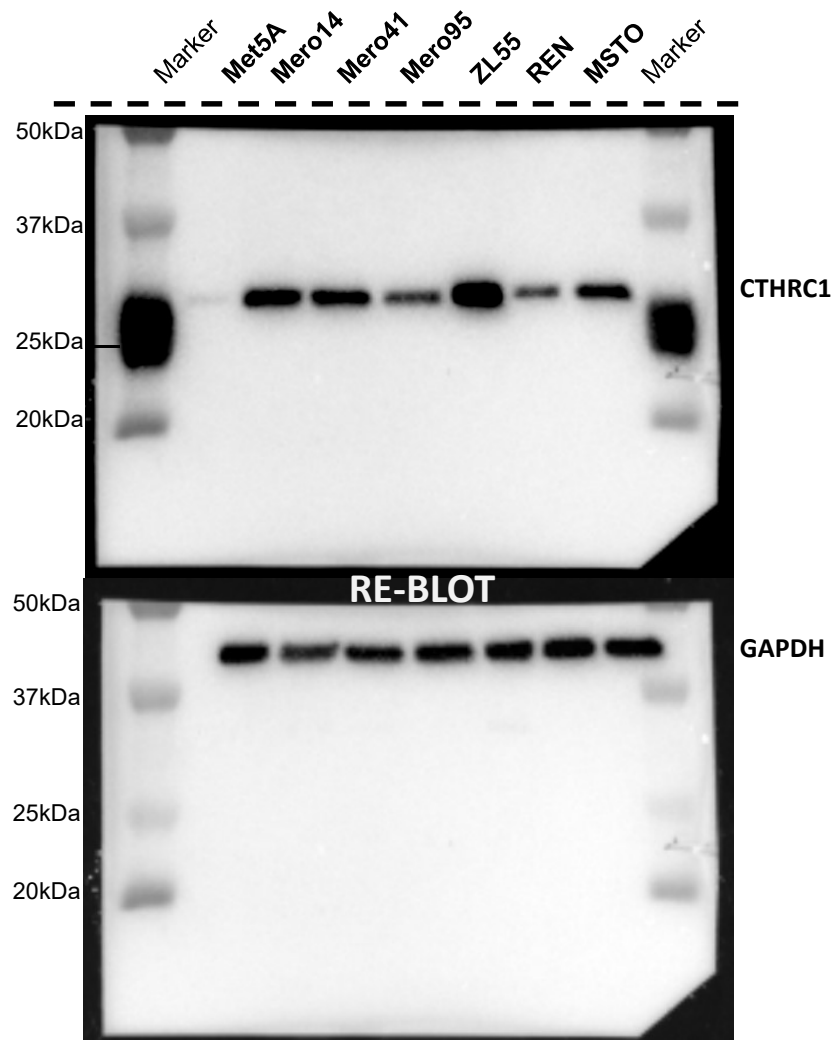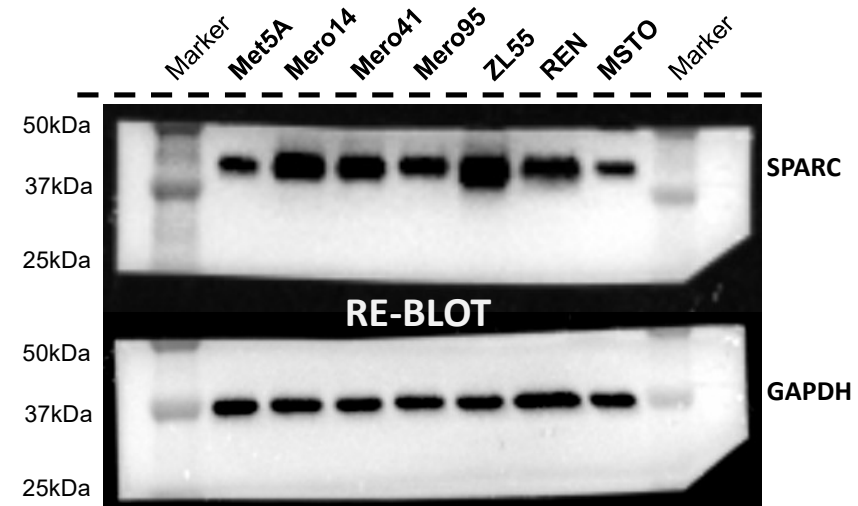

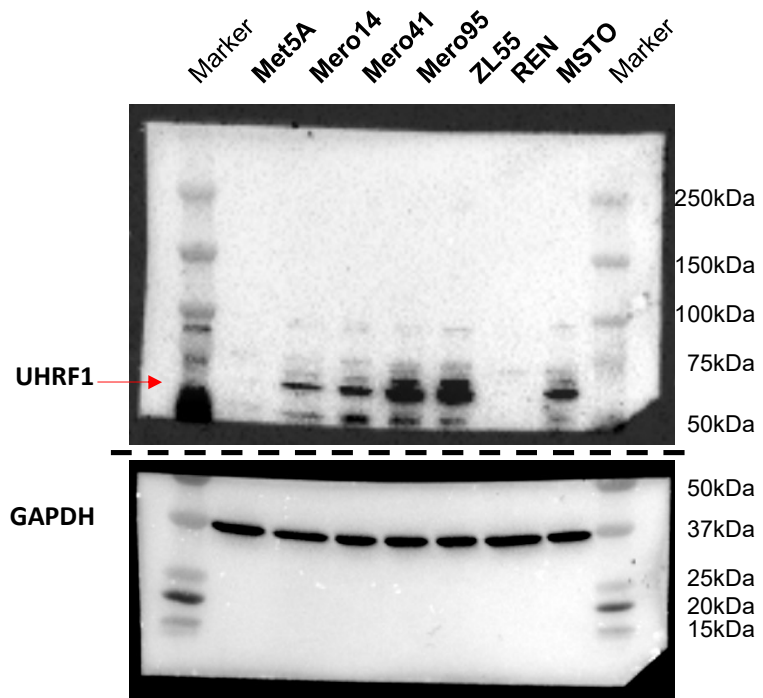

PRSS23

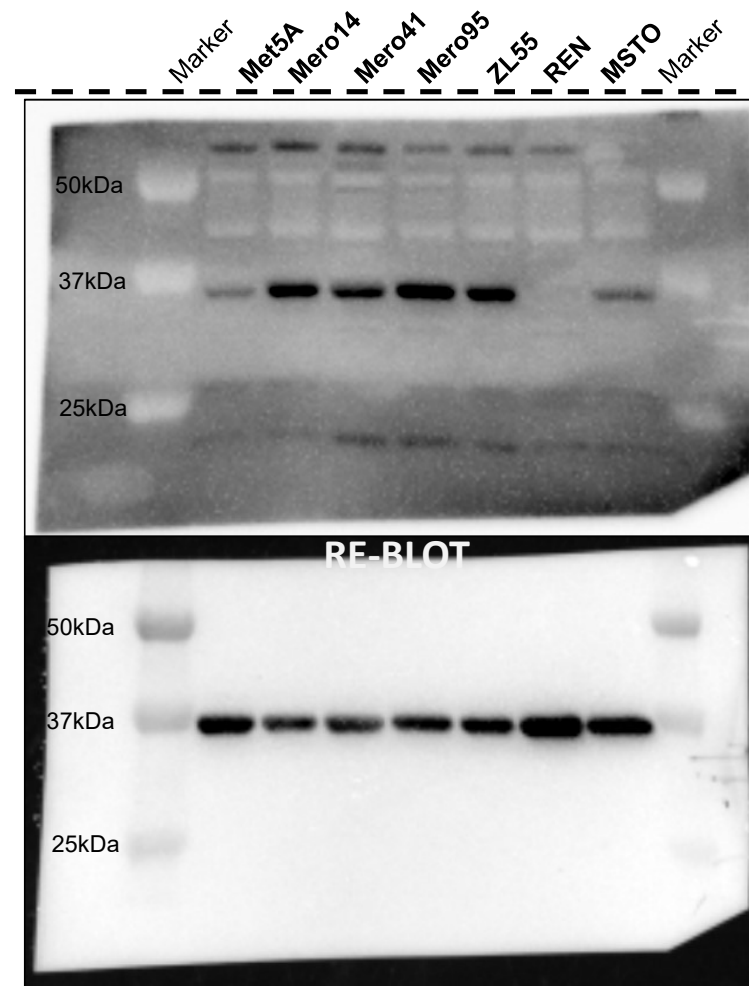

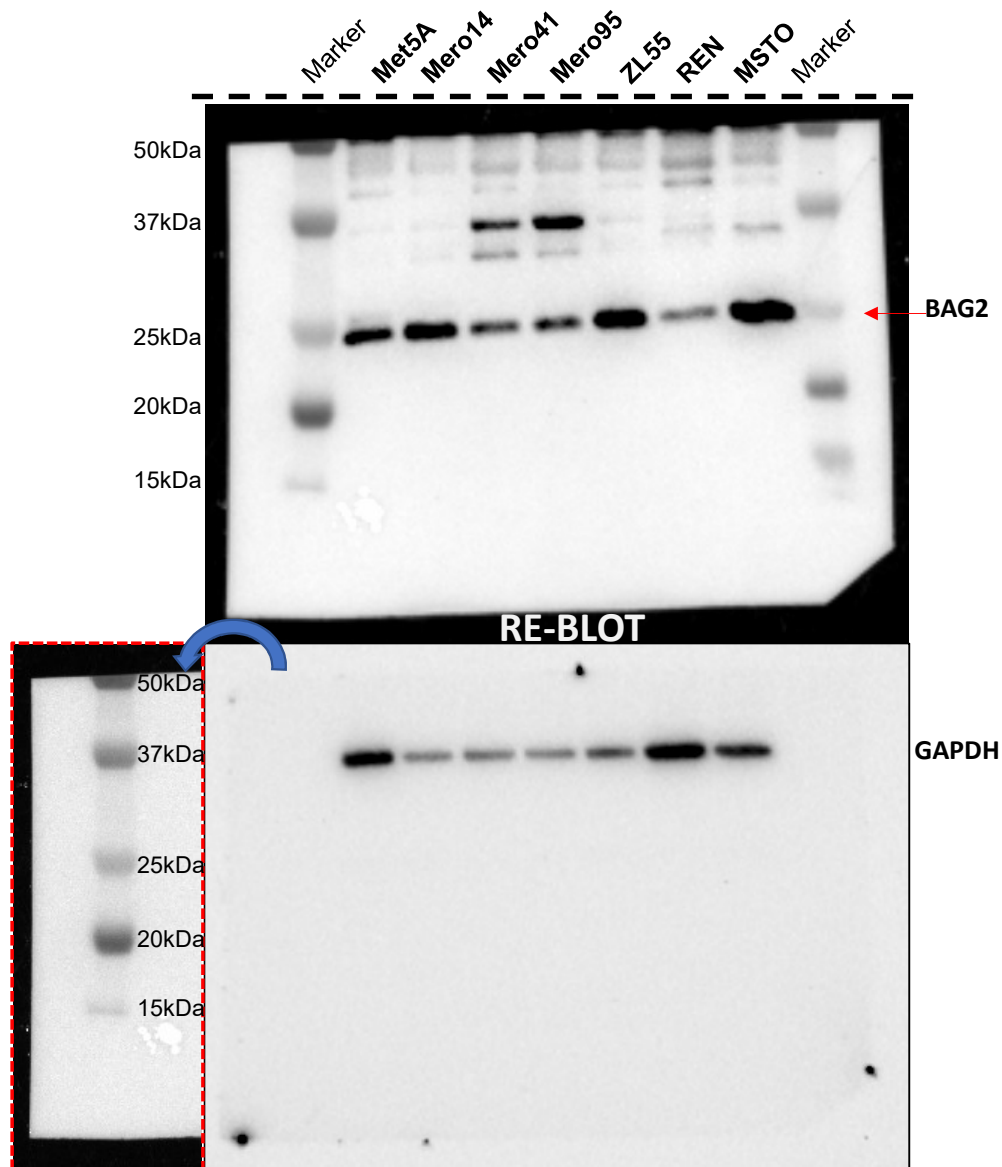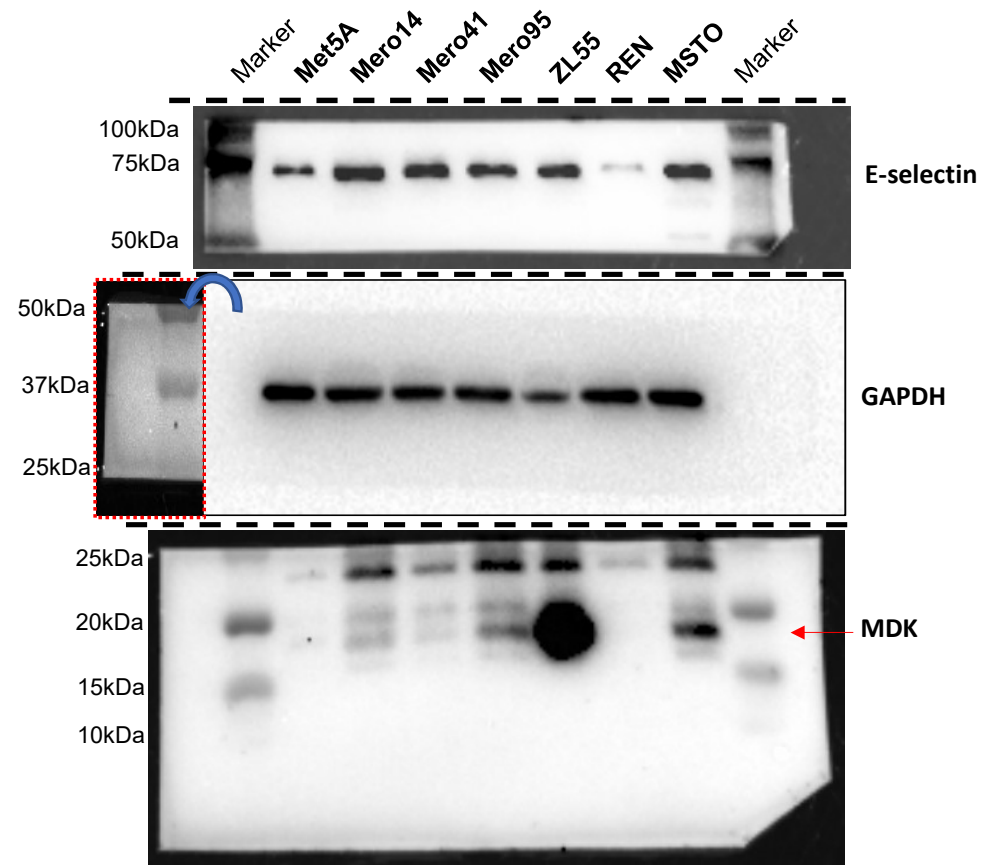

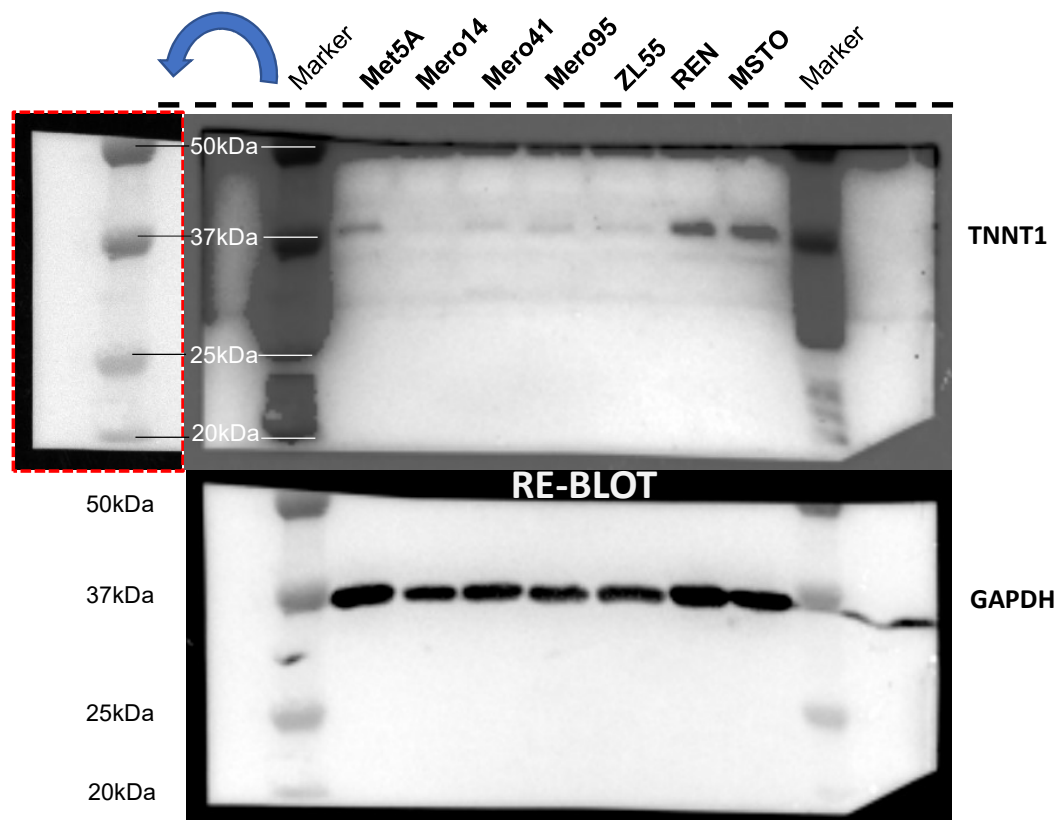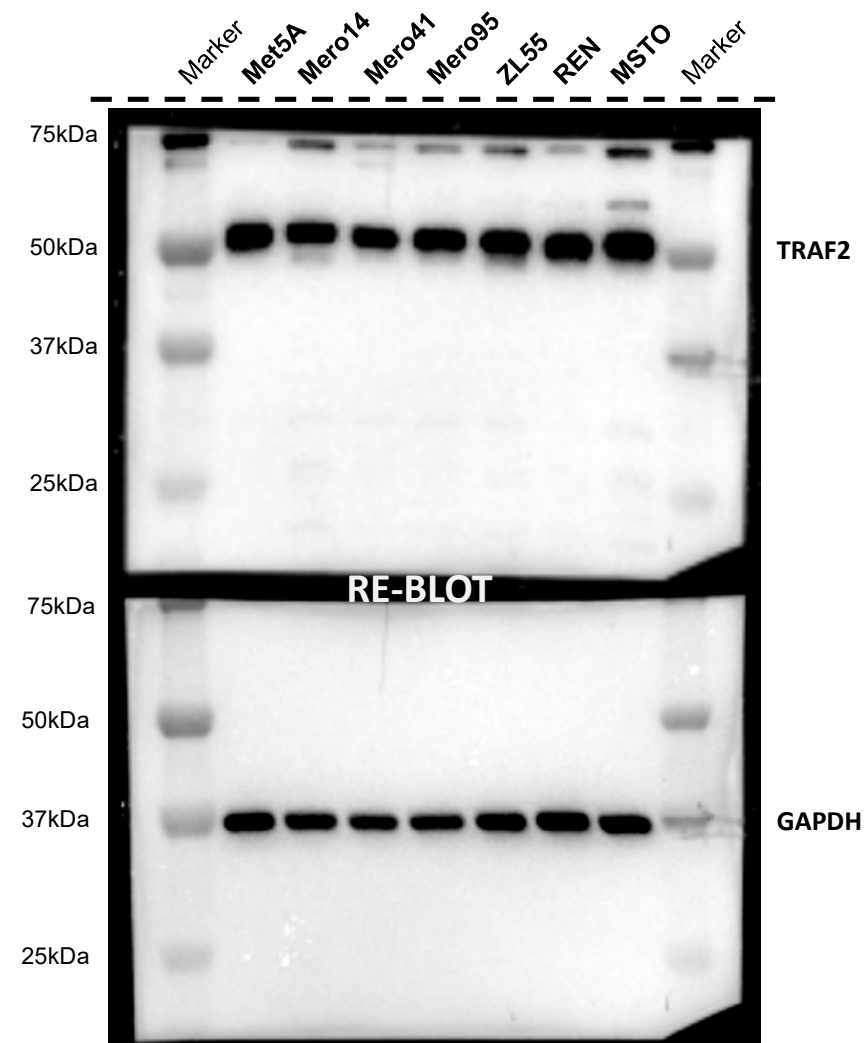



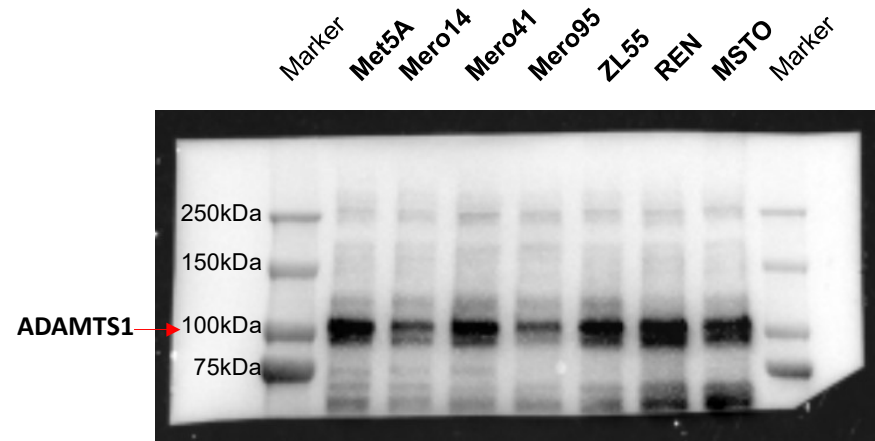

GAPDH

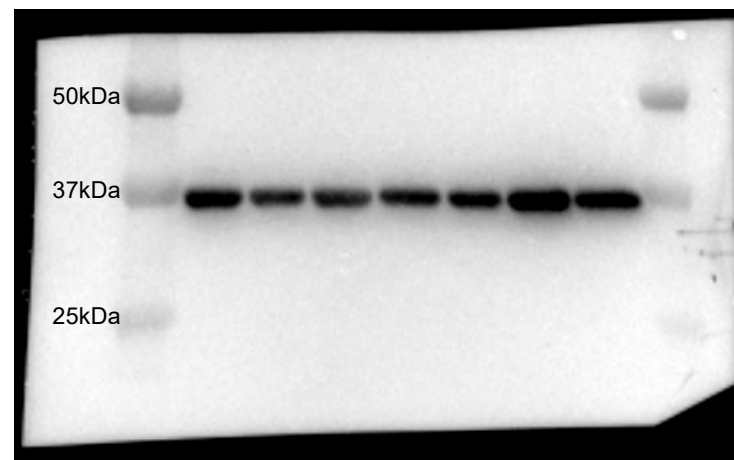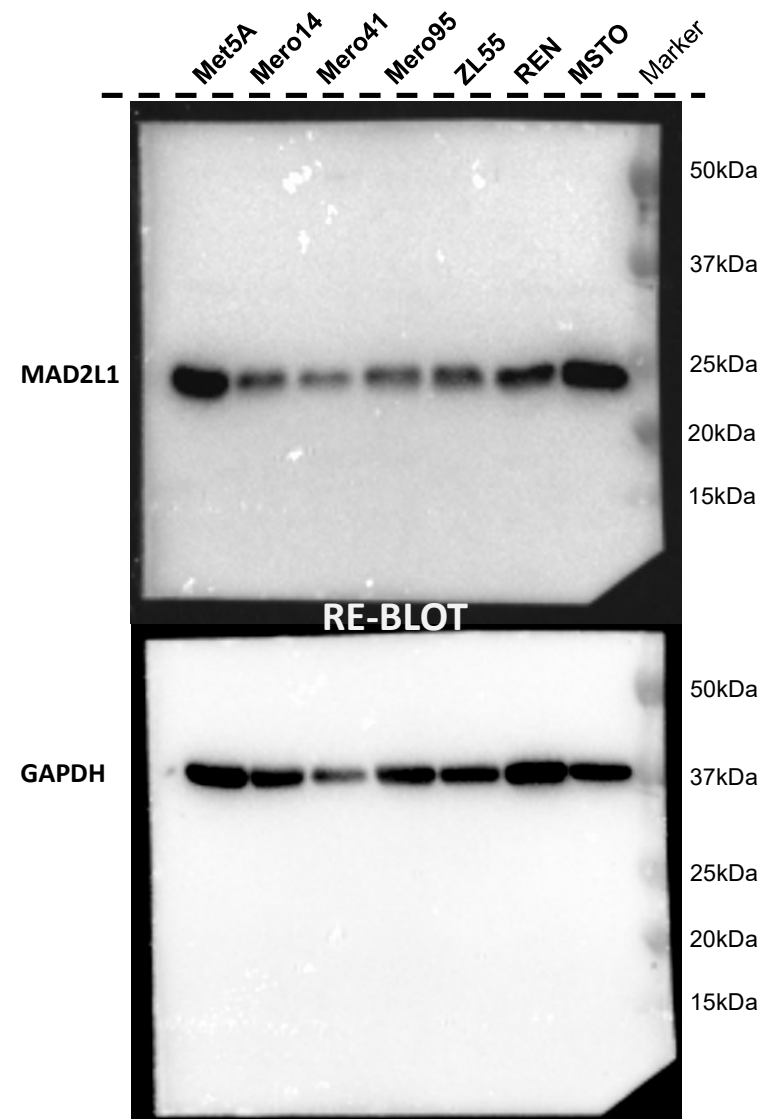

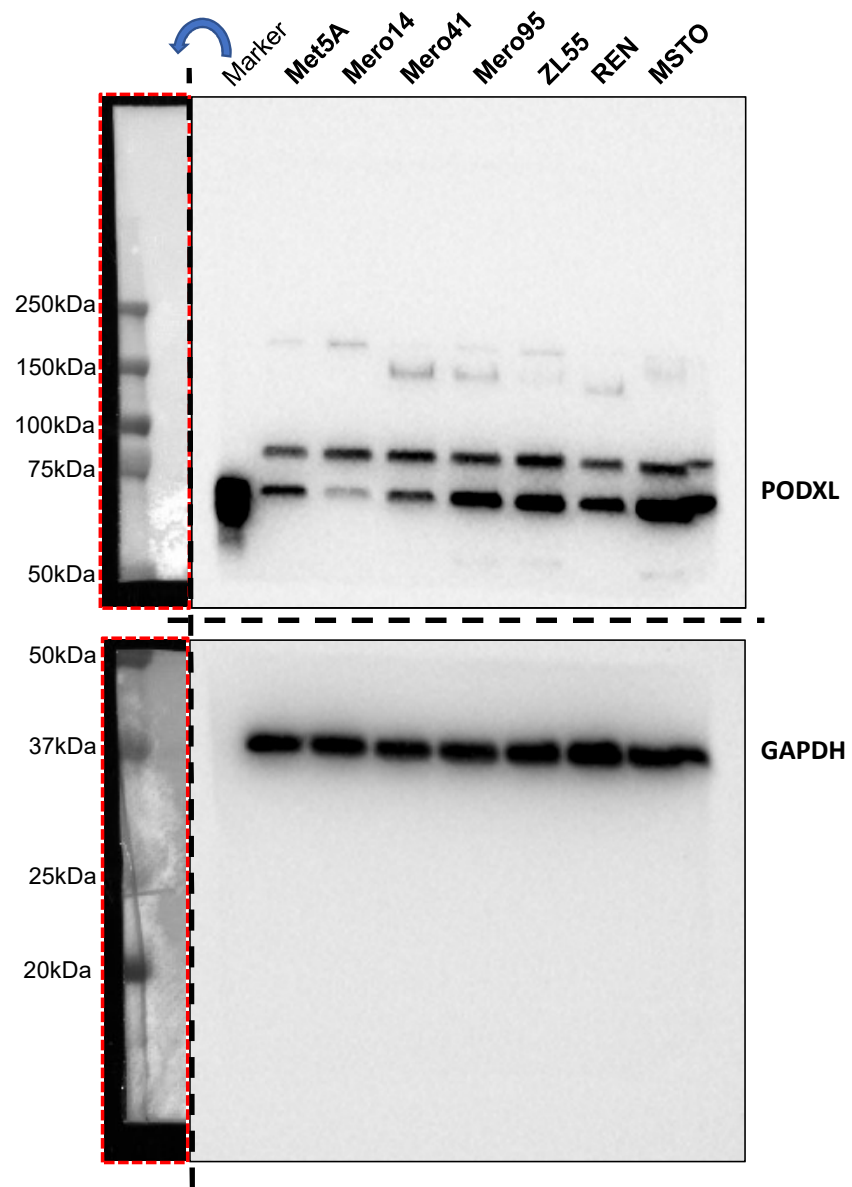

Supplement: Supplementary file 1 [file ijms-22-02738-s001.zip › Supplementary_final/Supplementary file 1.pdf]
